# Three Dimensional Cell Culturing for Modeling Adrenal and Pituitary Tumors

**DOI:** 10.3389/pore.2021.640676

**Published:** 2021-04-21

**Authors:** Lilla Krokker, Borbála Szabó, Kinga Németh, Rebeka Tóháti, Balázs Sarkadi, Katalin Mészáros, Attila Patócs, Henriett Butz

**Affiliations:** ^1^ Department of Laboratory Medicine, Semmelweis University, Budapest, Hungary; ^2^ Hereditary Tumours Research Group, Hungarian Academy of Sciences and Semmelweis University, Budapest, Hungary; ^3^ Department of Molecular Genetics, National Institute of Oncology, Budapest, Hungary

**Keywords:** 3D cell culture, adrenal tumor, adrenocortical carcinoma, pheochromocytoma, pitNET, pituitary adenoma

## Abstract

*In vitro* monolayer conditions are not able to reproduce the complexity of solid tumors, still, there is scarce information about the 3D cell culture models of endocrine tumor types. Therefore, our aim was to develop *in vitro* 3D tumor models by different methodologies for adrenocortical carcinoma (H295R), pituitary neuroendocrine tumor (RC-4B/C and GH3) and pheochromocytoma (PC-12). Various methodologies were tested. Cell biological assays (cell viability, proliferation and live cell ratio) and steroid hormone production by HPLC-MS/MS method were applied to monitor cellular well-being. Cells in hanging drops and embedded in matrigel formed multicellular aggregates but they were difficult to handle and propagate for further experiments. The most widely used methods: ultra-low attachment plate (ULA) and spheroid inducing media (SFDM) were not the most viable 3D model of RC-4B/C and GH3 cells that would be suitable for further experiments. Combining spheroid generation with matrigel scaffold H295R 3D models were viable for 7 days, RC-4B/C and GH3 3D models for 7–10 days. ULA and SFDM 3D models of PC-12 cells could be used for further experiments up to 4 days. Higher steroid production in 3D models compared to conventional monolayer culture was detected. Endocrine tumor cells require extracellular matrix as scaffold for viable 3D models that can be one reason behind the lack of the usage of endocrine 3D cultures. Our models help understanding the pathogenesis of endocrine tumors and revealing potential biomarkers and therapeutic targets. They could also serve as an excellent platform for preclinical drug test screening.

## Introduction

Experiments on *in vitro* cancer cell lines have contributed valuably in understanding the biology of cancers. *In vitro* conditions provide excellent setting to use numerous functional assays and it gives the advantage of modifying several variables and methods in controlled environments with easy handling, cost-effectiveness and good reproducibility [1]. However, *in vitro* monolayer conditions are not able to reproduce the complexity and three dimensional (3D) structure found in solid tumors [1, 2].

One sided adhesion induces a different polarity and cell-cell connections, and the absence of cell-extracellular environment matrix (ECM) interaction cannot mimic the natural structure of tissues. These factors result in differences, among others, in cell differentiation, gene expression and cellular metabolism processes [3].

In line with this, growing evidence points out the different efficacy between *in vitro* cell culture and *in vivo* (both xenograft and clinical trials) outcome based on *in vitro* anti-cancer drug screening experiments [1, 4]. These discrepancies highlight the need for more reliable *in vitro* cancer models.

A new era has started by the utilization of 3D cell culture techniques. 3D cultures can be categorized in two main classes: 1) non-scaffold based and 2) scaffold based 3D cell cultures [2]. 3D spheroids are non-scaffold based micro-sized cellular aggregates which are able to mimic various features of solid tumors, including cellular heterogeneity, cell-cell signaling, defined structure (composed of different cell layers), ECM deposition, ECM-cell and cell-cell physical interactions, growth kinetics, gene expression and drug resistance [2]. In scaffold-based 3D cultures, cells grow anchored to special material that mimics the ECM architecture providing more similar environment to biological situation. It is also described that ECM and stroma modify tumor cell behavior and response to treatment [5]. Drug resistance was repeatedly reported different in 2D and 3D cultures as cancer cells change their responsiveness to drugs by changing their interaction with their surroundings [1, 4]. Also, xenograft models display higher complexity but often do not predict human drug responses accurately due to species-specific differences [5]. The advantage of the *in vitro* 3D cell culture system is that it provides a well-controlled environment and it contributes to reducing the use of laboratory animal models referring cost and ethical issues.

Malignant (neuro)endocrine tumors are generally therapy-resistant exhibiting poor survival [6–8]. As they often resist to conventional therapies the development of a better 3D tumor model is needed to be used for further investigations and to be incorporated into research experimental design as in case of other malignancies. Interestingly, in several tumor types 3D models have been introduced into research modalities however they are lacking regarding endocrine tumor types. To date, four of each adrenocortical carcinoma, pituitary neuroendocrine tumor (pitNET) and pheochromocytoma [9–20] reports have been published using 3D cultures, they are different in terms of the applied methods and no viability and lifespan analysis have been tested and compared among different culture conditions. Therefore, in the use of 3D culturing in routine research applications the methodology should be optimized and standardized in order to maintain viability observed in *vivo* systems.

Therefore, in the current study our aim was to develop and optimize *in vitro* 3D tumor models by different methodologies for adrenocortical carcinoma, pituitary neuroendocrine tumor (pitNET) and pheochromocytoma using the commercially available cell lines.

## Material and Methods

### Cell Lines and Monolayer Culturing

NCl-H295R, PC-12, RC-4B/C and GH3 cell lines were selected to examine as representative neuroendocrine cancer cell lines. Cell lines were obtained from LGC Standards GmbH (Wesel, Germany) in the frame of LGC-ATCC partnership with a corresponding authentication certificate. Cells were propagated and freezed between one to four passages. They were used for experiments between 5–25 passages. Cells were maintained at 37°C and with 5% CO2 in T75 culture flasks with 10 ml appropriate medium as recommended by ATCC. H295R human adrenocortical carcinoma cell lines were grown in Dulbecco’s Modified Eagle Medium (10–013-CV, DMEM with 4.5 g/L glucose, l-glutamine, sodium pyruvate, Corning, Corning, NY, United States) supplemented with 10% fetal bovine serum (35–079-CV, FBS, Corning, Corning, NY, United States), 2.5% Nu serum (CB-51000, BD Biosciences, Franklin Lakes, NJ, United States), 1% ITS Premix universal Culture Supplement (354,350, Corning, Corning, NY, United States) and 1% penicillin/streptomycin (10,378,016, Thermo Fisher Scientific, Waltham, MA, United States). PC-12 rat adrenal medulla cell line was cultured in Ham’s F-2K medium with l-glutamine and sodium bicarbonate (N6658, Sigma-Aldrich, Merck, Kenilworth, NJ, United States) supplemented with 15% horse serum (16,050,122, Gibco, Thermofisher Scientific, Waltham, MA, United States), 2.5% FBS and 100 U/ml penicillin and 100 μg/ml streptomycin. RC-4B/C rat anterior pituitary adenoma cell line was maintained in Dulbecco’s modified Eagle’s medium and MEM (Minimum Essential Medium) Alpha Medium (10–022-CV, Corning, Corning, NY, United States) 1:1 ratio supplemented with 15 mM HEPES, 0.2 mg/ml bovine serum albumin (BSA, 15,561,020, Invitrogen, Thermofisher Scientific, Waltham, MA, United States), 2.5 ng/ml epidermal growth factor recombinant human protein (PHG0311, Gibco, Thermofisher Scientific, Waltham, MA, United States), dialyzed heat-inactivated fetal bovine serum (FBS) (26,400,044, Gibco, Thermofisher Scientific, Waltham, MA, United States), and 100 U/ml penicillin and 100 μg/ml streptomycin. GH3 rat anterior pituitary tumor cell line was grown in F12K medium (10–025-CV, Corning, Corning, NY, United States) supplemented with 10% FBS and 100 U/ml penicillin and 100 μg/ml streptomycin.

Culture medium was replaced with fresh complete medium in every 3 days. After cells reached 90% confluence they were dissociated from the bottom of the flask using 0.05% Trypsin-EDTA (25,300,062, Invitrogen, Thermofisher Scientific, Waltham, MA, United States). Imaging was done by Canon Power Shot A590 IS software using ×50 objective and ×10 ocular.

### Hanging Drop Cultures

Cells in 30 ul/drop were grown on the inside of the lid of 100 mm Petri dishes (CLS430167–20 EA, Corning, Corning, NY, United States). Different seeding cell numbers: 4.5 × 10^4^, 5.5 × 10^4^ and 6.5 × 10^4^ cells/drop were investigated.

### Ultra-Low Attachment Plate

Spheroid formation was induced by ultra-low attachment six well plates (3,471, Corning, Corning, NY, United States) with the optimal seeding densities in 2 ml medium: RC-4B/C and GH3 cell line 1.5 × 10^4^ cells/well; H295R cell line: 1.5 × 10^4^ cells/well; PC12: 1.8 × 10^4^ cells/well. After a 4-days process of spheroid induction cells were observed for seven more days under standard culture conditions. Cells were controlled daily by regular light microscopy.

### Application of Matrigel Matrix

For extracellular matrix mimicking Matrigel Matrix (354,262, Corning, Corning, NY, United States) was applied. For 3D spheroid culture two approaches were investigated. Cells were grown in matrigel or were layered on top of matrigel. Standard matrigel preparation was used. Briefly, all materials were chilled on ice to avoid premature gelation. 250 μL matrigel with 250 μL base media were mixed, then each well of a 6-well plate was coated with 500 μL diluted matrigel mixture. Matrigel matrix was then polymerized at 37°C for half an hour. 2 ml of complete medium including cells was layered on top of the matrigel. For RC-4B/C and GH3 cell line 1.5 × 10^4^ cells/well; H295R cell line: 1 × 10^6^ cells/well; PC12 cell line: 1.8 × 10^4^ cells/well were used. In the other approach cells were seeded in matrigel, preparing cell-medium-matrigel mixture with the same cell numbers. Cell Recovery Solution (354,253; Corning, Corning, NY, United States) was used to safety recover the cells, cultured on matrigel matrix for additional *in vitro* functional assays, following the manufacturers instructions. Briefly, after removing cell culture medium pre-chilled cell recovery solution was added in a volume of 2 ml (on 6-well plates/well). After 20 min incubation at 4°C the matrigel matrix was fully depolymerized. Then cells were scraped and they were centrifuged at 10,000 rpm for 5 min at 4°C, then the supernatant was discarded. Finally, cell pellets were washed with cold (4°C) PBS two times.

### Serum-free Defined Medium

For serum-free defined media basic cell culture medium recommended by ATCC according to each cell line type were applied. The general nutrient medium then was supplemented with 2% B-27 Supplement (17,504,001, Gibco, Thermofisher Scientific, Waltham, MA, United States), 50 ng/ml EGF (AF-100–15, PeproTech, Rocky Hill, NJ, United States) and 50 ng/ml basic-FGF (100–18B, PeproTech, Rocky Hill, NJ, United States).

### Cell Viability, Proliferation, Live-Dead Cell Ratio

Cells were seeded with different cell numbers per wells on each 6 well plates and cultured for multiple days (RC-4B/C: 150,000 cells/well; GH3: 150,000 cells/well; H295R: 250,000 cells/well; PC-12: 500,000 cells/well). For determining cell viability Alamar Blue assay (DAL1025, Invitrogen, Thermofisher Scientific, Grand Island, NY, United States), as a redox indicator was used. Fluorescent signals with excitation at 560 nm and emission at 590 nm were detected using a flash spectral scanning multimode reader (5,250,040, Varioskan, Thermofisher Scientific, Waltham, MA, United States) with SkanIt Software 2.4.5 RE. This assay is a resazurin-based solution that functions as cell health indicator by using the reducing power or metabolic activity of living cells to quantitatively measure viability. As viable cells continuously convert resazurin to resorufin the measured overall fluorescence represents the sum of viability of cells. Therefore, the results from Alamar Blue assays were considered as „viability”. Optical density (OD-s) data were presented as normalized values relative to monolayer cultures at each point in average ratio ± standard deviations. To investigate cell proliferation cell numbers were determined using 0.4% Trypan Blue staining (15,250,061, Gibco, Thermofisher Scientific, Waltham, MA, United States). Although Trypan Blue staining is used for investigating cell viability it represents a cruder analysis through identifying dead cells by staining (vs. metabolic well-being measured by Alamar Blue). Also, from the cell count we can assume proliferation more reliably. Therefore, results from Trypan Blue assays (live cell number) are defined as „proliferation”. For analyzing 3D models after spheroid formation, the cells were trypsinized then stained and analyzed. All measurements were done at least three times (biological replicates) with one to three technical replicates in each (total number of replicates for each conditions are indicated on each –). Mean and standard deviation were calculated. For comparison analysis of variance was used to identify statistical significance among different conditions, and Tukey's test was used to correct for multiple comparison in all cases. We evaluated each time point of each condition compared to 2D cultures *p* value <0.05 was considered as statistically significant.

### Steroid Hormone Measurements by HPLC-MS/MS

For LC-MS/MS analysis we used our previously published protocol with minor modifications adjusting for the usage of cell culture media [21]. Briefly, reference materials (cortisol solution 1 mg/ml dissolved in methanol and cortisone 250 mg) and the internal standard (certified reference material: 9,11,12,12-D4-cortisol 100 μg/ml solution, dissolved in methanol) were purchased from Sigma-Aldrich Hungary Ltd (Budapest, Hungary). LC-MS grade water, LC-MS grade methanol and LC-MS grade formic acid were purchased from VWR International Ltd (Debrecen, Hungary).

At the beginning of sample preparation 10 µL internal standard (2.76 μmol/L) was added to 90 μL cell culture media. Protein precipitation was carried out by adding 300 µL acetonitrile. After vortexing, samples were centrifuged for 5 min at 13,500 rpm. The supernatant was diluted in 1:1 proportion with LC-MS grade water after which the sample was ready for analysis.

LC-MS/MS assays were performed on a Perkin-Elmer Flexar FX10 ultra-performance liquid chromatograph coupled with a Sciex 5500 QTRAP mass spectrometer. For chromatographic separation a Phenomenex Kinetex C18 stationary phase column (50 × 2.1 mm, 1.7 µm) attached to a Phenomenex Security Guard Ultra C18 guard column (2 × 4.6 mm) was used (Gen-Lab Ltd., Budapest, Hungary). The mobile phase consisted of water containing 0.1% (v/v) formic acid (A) and methanol containing 0.1% (v/v) formic acid (B). The following gradient program was used: 10% B, hold 1.5 min, ramp B to 90% in 2 min, hold 1.5 min, ramp B to 10% in 0.5 min, hold 2.5 min. The total run time was 8.0 min, the flow rate was 200 μL/min and 20 µL sample was injected. Column temperature was set at 35°C. Elution peaks appeared at 5.50 min for cortisone and 5.60 min for cortisol and internal standard.

The mass spectrometer was operating in negative electrospray ionization mode with the following settings: source temperature: 350°C, ionization voltage: 4500 V, curtain gas: 35 psi, gas1: 35 psi, gas2: 35 psi, entrance potential: 10 V, CAD gas: medium.

Quantitative analysis was performed in multiple reaction monitoring (MRM) mode, with the following compound specific settings: cortisol quantifier ion 407.215→331 (declustering potential (DP): 55 V, collision energy (CE): 22 V, cell exit potential (CXP): 21 V), cortisol qualifier ion 407.215→282 (DP: 55 V, CE: 31 V, CXP: 27 V), cortisone quantifier ion: 405.21→328.9 (DP: 35 V, CE: 20 V, CXP: 21 V), cortisone qualifier ion: 405.21→137 (DP: 35 V, CE: 48 V, CXP: 9 V), internal standard quantifier ion 411.2→335.2 (DP: 60 V, CE: 31 V, CXP: 27 V) and internal standard qualifier ion 411.2→301.2 (DP: 60 V, CE: 45 V, CXP: 24 V) [21].

## Results

We investigated 3D cell cultures generated with different methods and compared them to conventional 2D (monolayer) environment. We used hanging drop, classical spheroid inducing media (serum-free defined media, SFDM), ultra-low attachment plate and matrigel to mimic extracellular matrix.

In hanging drop cultures cells formed cell aggregates however media change and cell propagation were cumbersome and difficult not making spheroid formation convenient and easy handling for further downstream experiments. Also, hanging drop due to suspension culture was difficult to visualize, therefore we omitted to use this method in our further investigation.

Regarding matrigel usage we also investigated cells embedded in matrigel and layered on top of matrigel. Recovering cells completely embedded in matrigel matrix required more digestion affecting cell viability. It was more laborious, and making 3D model preparation in standardized condition for further experiments was more challenging. Also, cells embedded in matrigel due to being thick layer were difficult to visualize. As both embedding in and layering 3D cultures on matrigel showed similar spheroid morphology, we selected 3D cultures layering on matrigel matrix for further investigations.

### Adrenocortical Carcinoma *In Vitro* 3D Models

**FIGURE 1:**
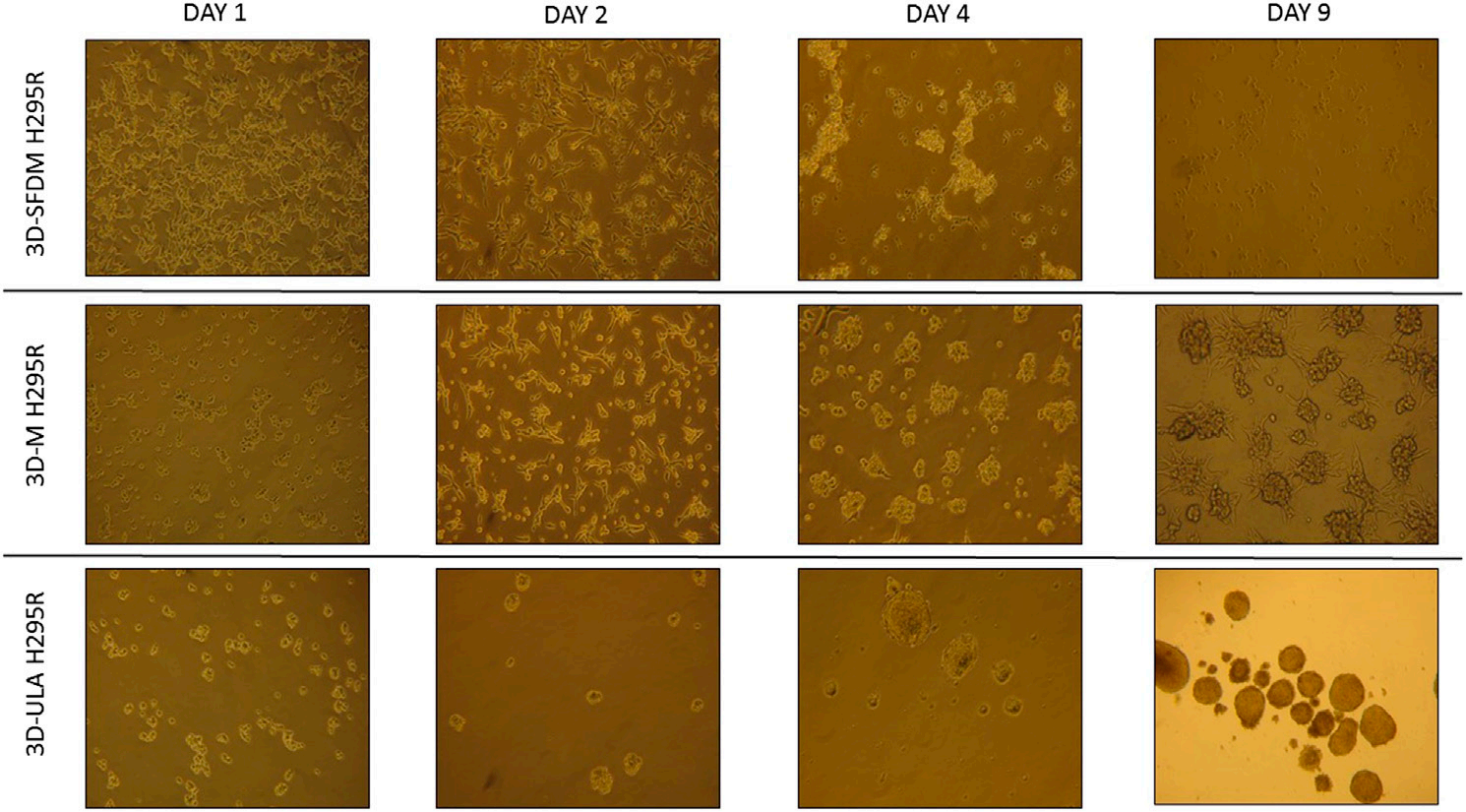
H295R (adrenocortical carcinoma) cells’ phase contrast images of three dimensional (3D) cultures taken on day 1, 2, 4 and 9. Imaging was done by ×500 magnification (see methods). 3D-SFDM: 3D model generated using serum-free defined media; 3D–M: 3D model generated by martigel matrix; 3D-ULA: 3D model generated by ultra-low attachment plate.

**FIGURE 2:**
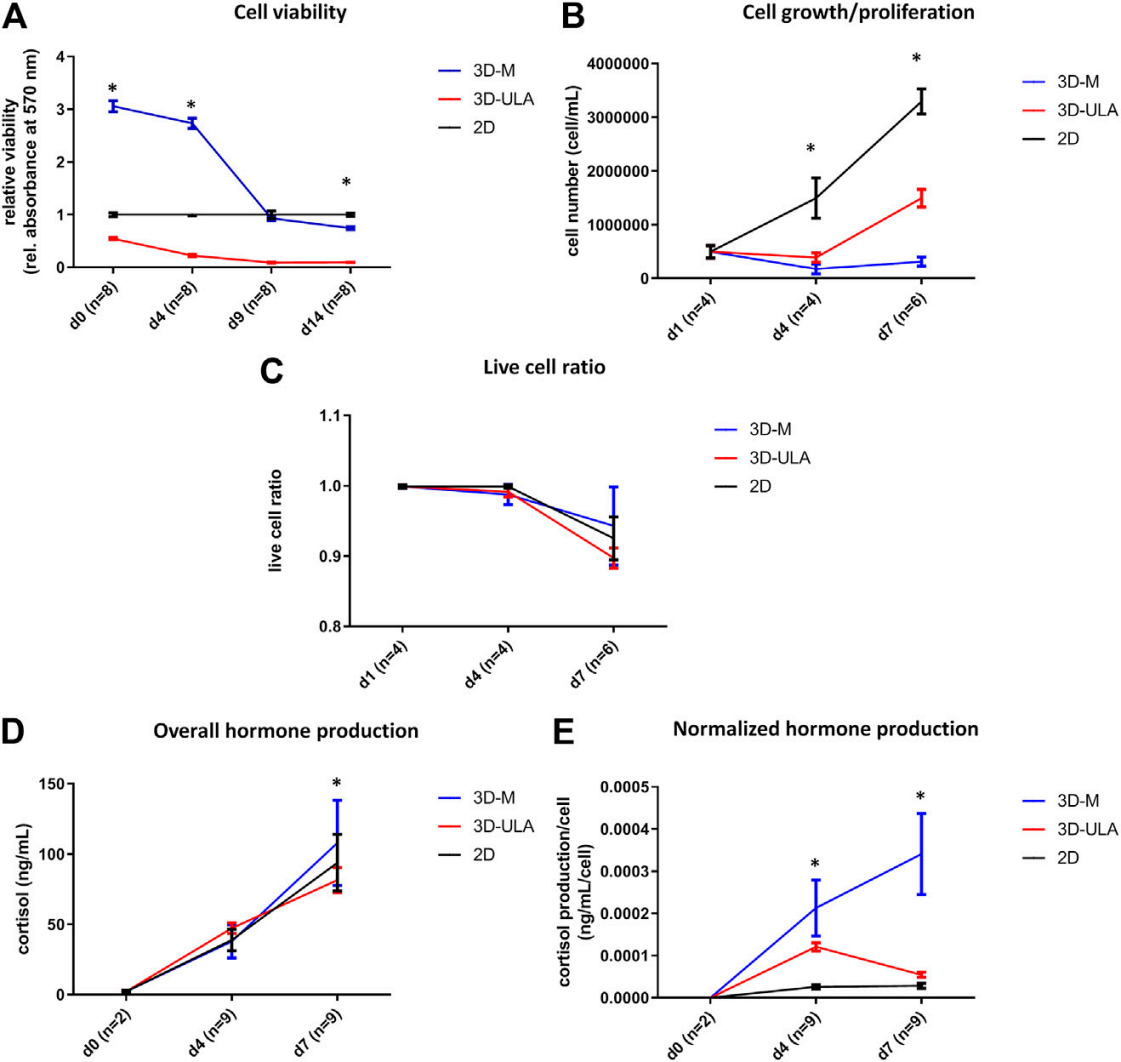
*In vitro* viability **(A)**, proliferation **(B)**, live cell ratio **(C)** and hormone production **(D,E)** of different H295R 3D culture models. 3D-SFDM: 3D model generated using serum-free defined media; 3D–M: 3D model generated by martigel matrix; 3D-ULA: 3D model generated by ultra-low attachment plate; **D**: day; n: number of replicates. Stars indicate statistical significance (*p* < 0.05) as follows: **A**: 3D-ULA, 3D-M vs. 2D at d0, d4, d14. 3D-M vs. 3D-ULA at each time point. **B**: 3D-ULA, 3D-M vs. 2D at d4, d7. 3D-M vs. 3D-ULA at d7. **C**: no statistical significance. **D**: 3D-ULA vs. 3D-M at d7. **E**: all 3D-M vs 2D and 3D-M vs 3D-ULA at d4 and d7. 3D-ULA vs 2D at d4.

We investigated H295R 3D cell cultures generated by SFDM (3D-SFDM), ultra-low attachment (ULA) plate and matrigel (3D-M). Approximately 2–4 days were required for multicellular spheroid formation (Figure 1). Although SFDM is widely used for spheroid induction, H295R adrenocortical carcinoma cell line did not only refuse to form 3D structures but as it seen on Figure 1, but rapid cell degradation was experienced with increasing floating proportion during day 2–4 evolving into complete cell death. Therefore, we omitted to perform viability and proliferation assays in this condition. Using ultra-low attachment plate (3D-ULA model) in standard medium was a less preferred condition for Adrenocortical carcinoma (ACC) cells compared to monolayer culturing or 3D-M in terms of cell viability, proliferation and live cell ratio (Figures 2A–C). Also, the 3D culture in extracellular matix (3D-M) showed the highest live cell ratio (and the lowest dead cell ratio) (Figure 2C). As expected, cells in 3D environment performed slower proliferation rate compared to 2D cells mimicking the *in vivo* conditons. In ACC the only available medication is mitotane, an adrenolytic treatment decreasing hormone production. Hence, we also investigated cortisol secretion of the different models (Figures 2D,E). Surprisingly, after normalizing hormone production to cell number, both 3D models showed increased hormone secretion compared to monolayer culture, however 3D-M cultures stayed viable and demonstrated continously increasing steroid production compared to 3D-ULA model (Figure 2E). The normalized hormone production also showed that in 3D-ULA model after day 4 no additional hormone secretion occurred (Figure 2E). Based on our results, 3D-M model can be used up to 7 days for further experiments without major decrease of live cells (Figure 2C).

### PitNET *In Vitro* 3D Models

**FIGURE 3:**
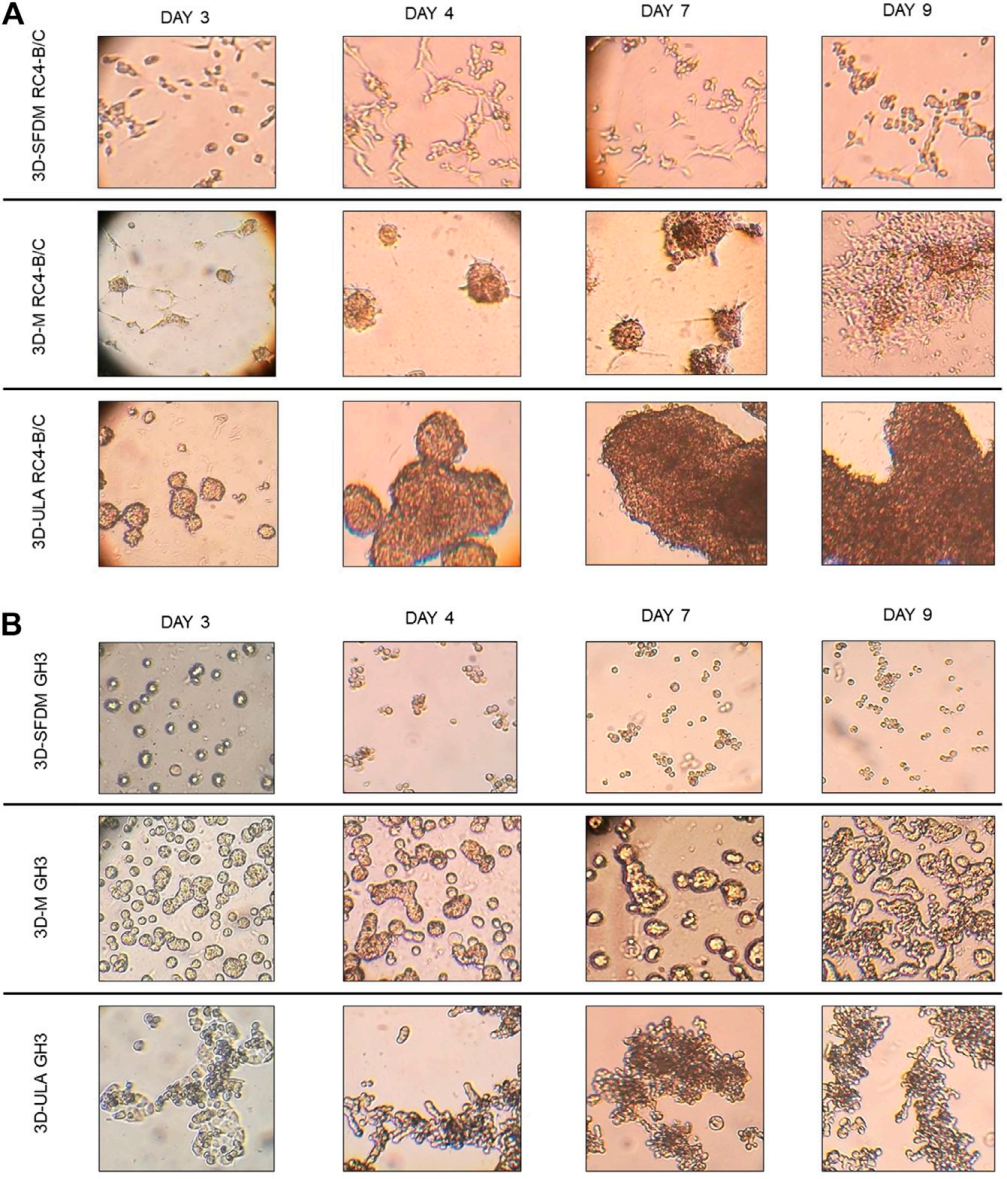
RC-4B/C **(A)** and GH3 **(B)** pituitary cells’ phase contrast images of 3D cultures taken on day 3, 4, 7 and 9. Imaging was done by ×500 magnification (see methods). 3D-SFDM: 3D model generated using serum-free defined media; 3D–M: 3D model generated by martigel matrix; 3D-ULA: 3D model generated by ultra-low attachment plate.

**FIGURE 4:**
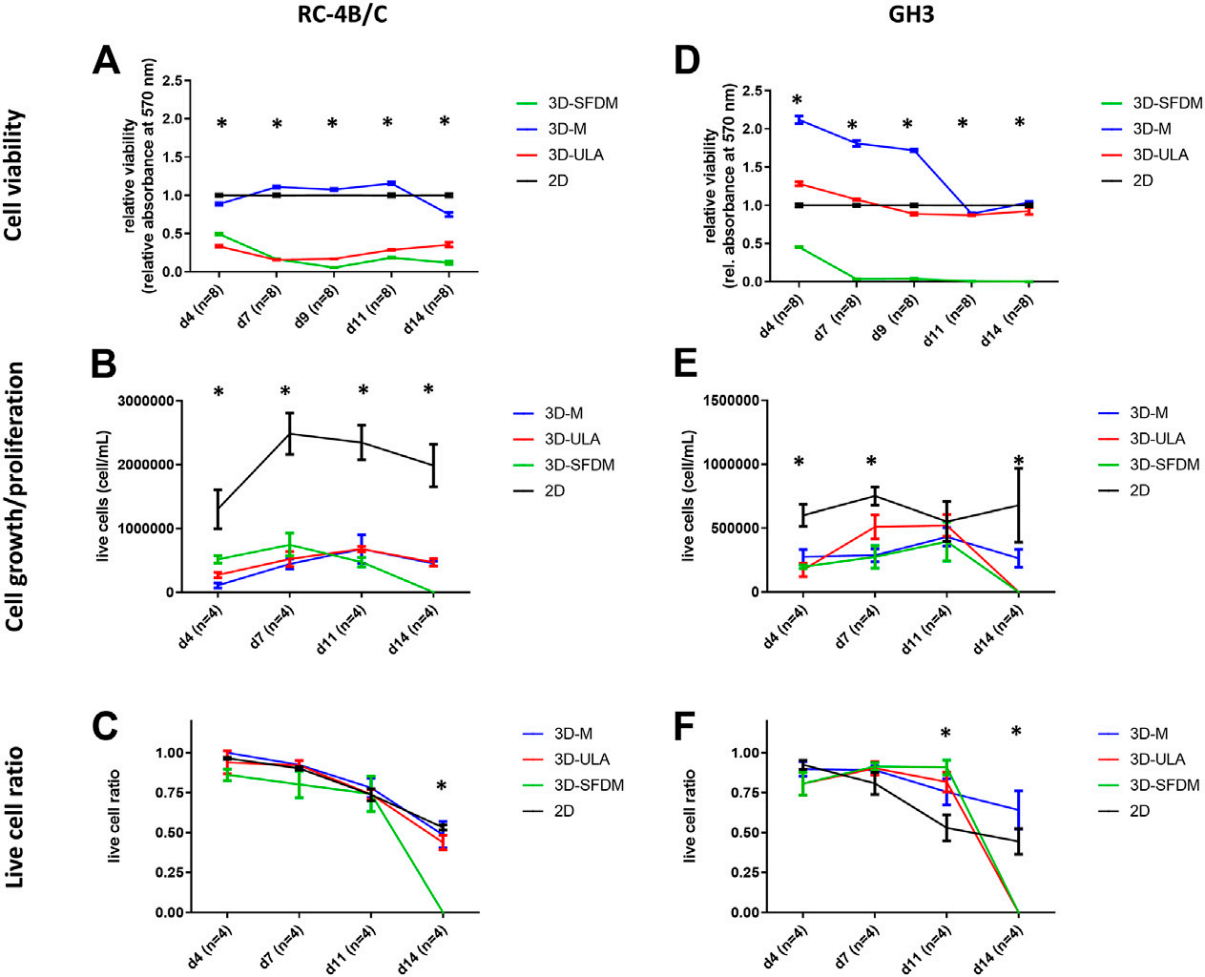
Functional *in vitro* assays of different 3D culture models generated from RC-4B/C **(A–C)** and GH3 cells **(D–F)**. 3D-SFDM: 3D model generated using serum-free defined media; 3D–M: 3D model generated by martigel matrix; 3D-ULA: 3D model generated by ultra-low attachment plate; **D**: day; n: number of replicates. Stars indicate statistical significance (*p* < 0.05) as follows: **A**: 3D-ULA, 3D-SFDM vs. 2D. **B**: 3D-M, 3D-SFDM, 3D-ULA vs. 2D. **C**: 3D-SFDM vs. all other conditions. d: 3D-M, 3D-SFDM, 3D-ULA vs. 2D at d4, d9, d11, d14. 3D-M, 3D-SFDM vs. 2D at d7. e: all 3D conditions vs. 2D at d4 and d14. 3D-M, 3D-SFDM vs. 2D at d7. f: all 3D conditions vs. 2D at d11 and d14.

In the lack of human pituitary tumor cell lines, GH3 and RC-4B/C rat pituitary cell lines were used for 3D model generation (Figures 3A,B). The plurihormonal RC-4B/C cells did not form 3D structures in SFDM, moreover they exhibited low viability using metabolic assay (Figure 4A), however by counting live and dead cells the live cell ratio dropped only after 11 days (Figure 4C). On ULA plates although cells formed small spheroids after 3 days but they showed low viability similarly to SFDM condition (Figure 4A) possibly due to producing massive cell conglomerates where oxygen and nutrient diffusion can be limited. RC-4B/C 3D-M pitNET model was viable and usable for further experiments for 7–10 days (Figures 4A–C). Similarly to RC-4B/C cells, using GH3 cell line 3D-M model was the best option compared to the other methods by morphological observation and SFDM was especially unfavorable environment for GH3 cells (Figure 3B). And however, the basic description of GH3 cells in monolayer environment is “loosely adherent with floating clusters” (ATCC) they did not favor ultra-low attachment plate either (they exhibited lower live and higher dead cell ratio compared to 2D monolayer culture) (Figures 3, 4F). Using matrigel matrix for 3D-M model generation the dead cell ratio was even smaller compared to the conventional monolayer culture (Figure 4F). The GH3 3D-M model can be used up to 9 days for further experiments.

### Pheochromocytoma *In Vitro* 3D Models

**FIGURE 5:**
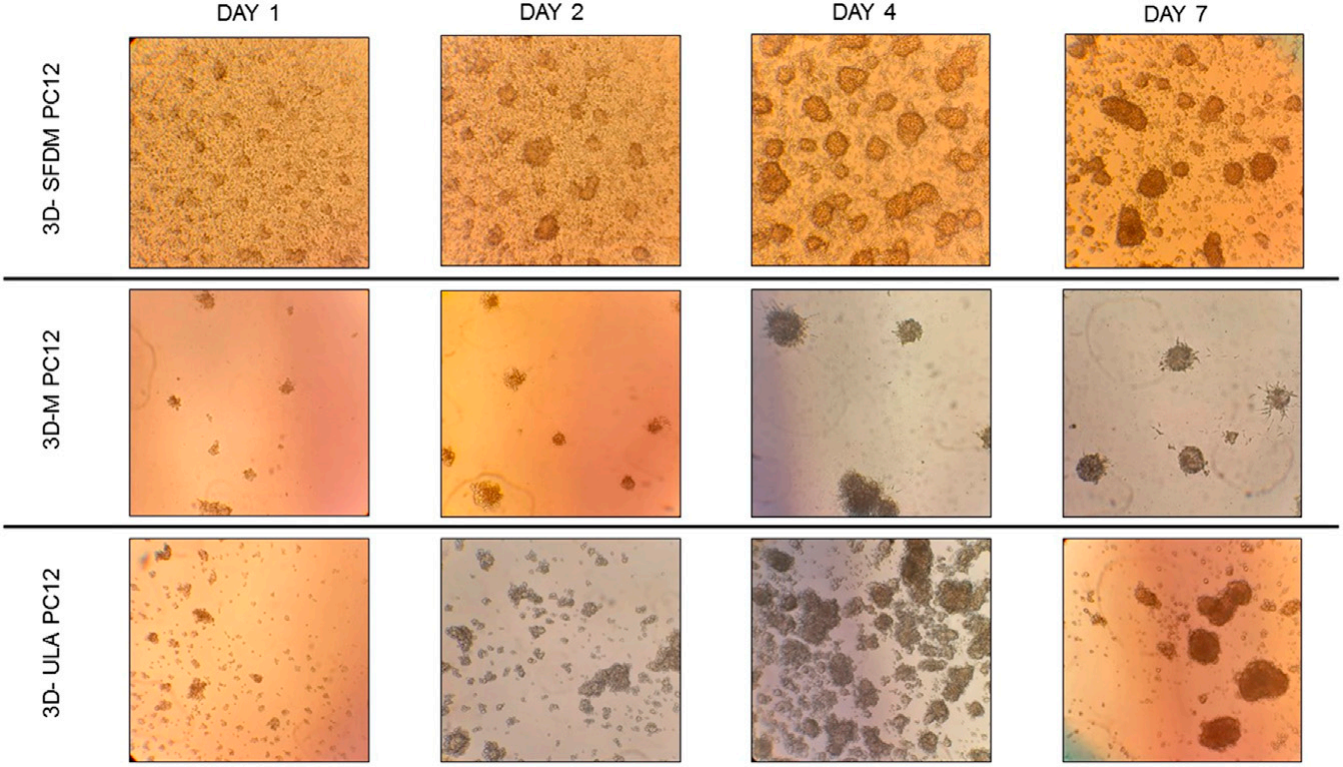
PC-12 rat pheochromocytoma cells’ phase contrast images of 3D cultures taken on day 1, 2, 4 and 7. Imaging was done by ×500 magnification (see methods). 3D-SFDM: 3D model generated using serum-free defined media; 3D–M: 3D model generated by martigel matrix; 3D-ULA: 3D model generated by ultra-low attachment plate.

**FIGURE 6:**
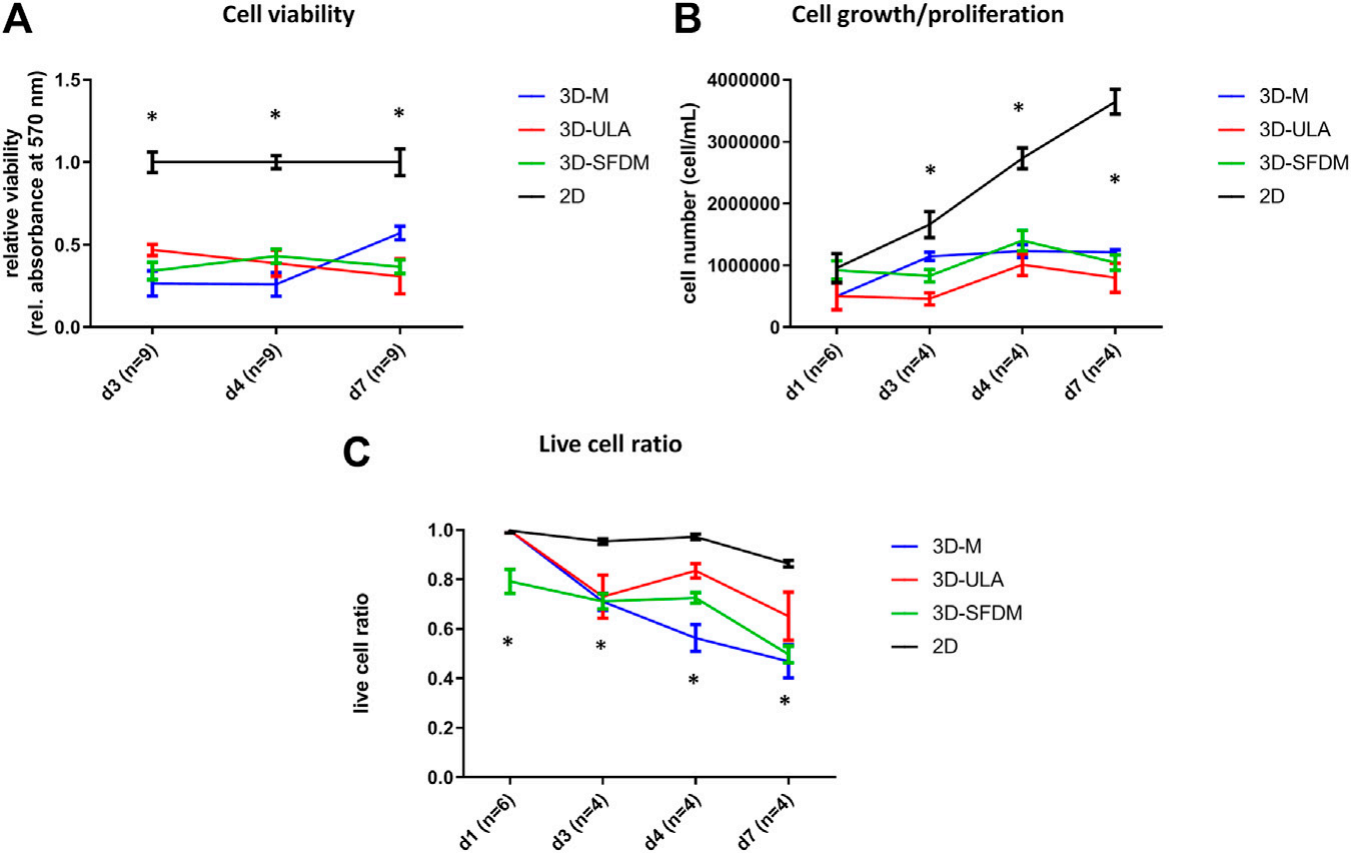
Functional *in vitro* assays of different PC-12 3D culture models: **(A)** viability, **(B)** proliferation and **(C)** live cell ratio. 3D-SFDM: 3D model generated using serum-free defined media; 3D–M: 3D model generated by martigel matrix; 3D-ULA: 3D model generated by ultra-low attachment plate; **D**: day; n: number of replicates. Stars indicate statistical significance (*p* < 0.05) as follows: **A**: all 3D conditions vs. 2D at each time point. **B**: all 3D conditions vs. 2D at d3, d4, d7. **C**: all 3D conditions vs. 2D at d3, d4, d7. 3D-SFDM vs. all other conditions at d1.

To study human pheochromocytoma, the rat PC-12 cell line is the most widely used *in vitro* model due to the lack of human cell line. Compared to ACC and pitNET cells pheochromocytoma cells formed *in vitro* 3D structures using all SFDM, ULA and matrigel matrix (Figure 5). However, while 3D-ULA model showed slightly decreasing viability with time, 3D-M PC-12 model on matrigel exhibited increasing viability (Figure 6A). Expectedly, cell growth is halted in 3D models compared to monolayer culture together with decreasing live cell ratios especially following day 3 (Figures 6B,C). Although morphologically 3D-M modell provided the most consistent structures, ULA seems to be the best method in terms of live cell ratio with slightly decreasing viability with time compared to 3D-M model (Figure 6C). 3D-ULA and 3D-SFDM PC-12 models could be used for further experiments up to 96 h due to decreasing live cell ratio after day 4 (Figure 6C).

## Discussion

In the last few years, three-dimensional culturing has emerged as a new field in research. A wide variety of methodologies are already available based on published data and manufacturer recommendations [3, 22]. There is a considerable difference between tumor cell behavior in cell cultures and solid tumors because monolayer cultures do not represent the heterogeneity and complexity of human tumors reliably [1, 4]. Treatment options of endocrine tumors are limited as they are frequently chemoresistant and do not respond to radiation therapy either [23]. Until now, minimal information about 3D culturing of endocrine tumors has been published.

The purpose of our research was the establishment of 3D *in vitro* models of adrenal and pituitary tumors and their comparison with monolayer cell cultures. The development of three-dimensional techniques for four selected neuroendocrine cell lines (NCl-H295R human adrenal carcinoma cell line, PC-12 rat adrenal chromaffin cell line, RC-4B/C and GH3 rat hypophysis adenoma cell line) has been performed. Successful spheroid constructions were generated from all cell lines using at least one of the tested methods. We determined the optimal culture environments and also the viable life-span of 3D models for each cell type. After setting up the optimal culture conditions these cultures can be further propagated by trypsinization. One common limitation of these 3D methods is the shorter application time compared to 2D cultures resulting from the avascular nature of 3D structures. However, by regular trypsinization they can grow continually in the appropriate 3D environment. Interestingly, we found, that unlike other cancer types, endocrine tumor types do not prefer spheroid inducing media (SFDM) and ultra-low attachment (ULA) plates which are the two most commonly used methods presented to date. Rather, they require extracellular matrix for viable 3D structures making *in vitro* culturing and propagation more complicated compared to the use of ULA and SFDM. It is possible that this has been one reason behind lacking 3D endocrine tumor models in literature. Here, we introduced an innovative and relatively easy technique combining spheroid formation with scaffold-based 3D model. In these models, cells remained viable and proliferating at a slower rate compared to their 2D counterparts.

In general, neoplasms of the endocrine system are considered chemoresistant and there are no available efficient anti-cancer treatment options (except in cases when somatostatin analogues can be administered). Adrenocortical carcinoma (ACC) is an aggressive neoplasm with dismal prognosis, and it often appears as metastatic disease at diagnosis [24]. Being a chemoresistant tumor, therapeutic options are limited [24]. Mitotane, an adrenolytic drug, has been the only anticancer-agent approved for treatment of ACC in adjuvant and palliative settings for many years. On mitotane treatment 20.5% objective response rate was observed with only 4.1-month progression free (PFS) and 18-months overall survival (OS) [24]. Therefore, our innovative 3D model can be beneficial in revealing ACC pathogenesis and potential new biomarkers. In patients with ACC, cortisol production has an exceptional clinical role: it has been described as negative prognostic factor, and the adrenolytic adjuvant mitotane therapy decreasing hormone secretion is the sole available targeted therapy [25]. Hence, the increased cortisol production of H295R 3D-M model compared to other models has a unique relevance for further investigations. Our result is reinforced by the results of Lichtenauer et al. 2013, which focused on the aldosterone production as a model of hyperaldosteronism in relation to different H295R culture conditions [26]. In their work, they also examined the difference between monolayer and spheroid cells in terms of aldosterone production. In their study spheroid cells were established using serum-free medium, they also reported higher aldosterone level concentration normalized to total cellular protein and also higher expression levels of the steroidogenic enzymes (StAR, 3βHSD, CYP17, SF-1, and the MC2-receptor) [26].

Pituitary adenomas, although the vast majority of them are benign, especially non-functional tumors can be recognized late when involving surrounding sinuses making impossible the complete surgical resection [27]. These refractory pitNETs are usually resistant to regularly used dopamine agonists and somatostatin analogs [27]. Additionally, most pitNET are sporadic and apart from a couple cases where somatic mutations are identified, there is no clear molecular background known behind tumorigenesis [28]. Comprehensive 3D model of pitNET has not been published, however, successful GH3 spheres have been already generated using hanging drop technique or surface-treated microplate [14, 29]. Ours is the first report of successful spheroid induction on the RC-4B/C cell line. Therefore, our 3D culture characterization can be useful in understanding pitNET pathogenesis and revealing potential therapeutic targets in refractory pitNETs.

Malignant (metastatic) pheochromocytoma is a very rare condition and it carries a poor prognosis with a 5-years survival rate of 44% [30]. Most pheochromocytoma cases are sporadic, but 30–40% of all cases is associated with germline mutations and hereditary syndromes. Among these carrying germline *SDHB* mutation is currently the most important contributor to hereditary malignant form [31]. Significant knowledge is available regarding pheochromocytoma pathogenesis maily involving hypoxia inducible one alpha signaling [32, 33] but currently there has been no systemic therapy approved [34]. Additionally, there is no validated pathology criteria for determining whether a primary pheochromocytoma is beingn or malignant [35]. Therefore, 3D culture models can help to evaluate our current knowledge derived from conventional monolayer cultures and also can help to screen the drugs as potential targets. Using pharmacological inhibition of SDHB by itaconate and concomitant inhibition of glutaminase in 3D-SFDM cultured PC12 cells significant decrease of living cell ratios compared to vehicle treatment was observed, further supporting that this model can be successfully used in evaluation of novel therapeutical option in this disease [9].

As a conclusion, we developed an innovative, viable 3D model system of adrenal and pituitary tumors combining spheroid formation with matrigel scaffold. To the best of our knowledge this is the first report comparing different methods for *in vitro* 3D endocrine tumor models and evaluating their life-span and viability. Our results suggest, that compared to other tumor types, adrenal and pituitary tumor cell types require extracellular matrix as a scaffold for a viable model to avoid molecular genetic and metabolic changes due to hypoxic environment and in the lack of nutrients in simple multicellular aggregates. These models allow reevaluation of the findings derived from monolayer cultures, the understanding neuroendocrine tumor pathogenesis and revealing potential new biomarkers and therapeutic targets. These models could also serve a basis for preclinical drug test screening.

## Data Availability

The original contributions presented in the study are included in the article/Supplementary Material, further inquiries can be directed to the corresponding author.
